# Giving Voice to Cardiovocal Syndrome: A 26-Year-Old Woman With Hypophonia and Dysphagia

**DOI:** 10.7759/cureus.48917

**Published:** 2023-11-16

**Authors:** Kristen A Ehrenberger

**Affiliations:** 1 Internal Medicine, University of Pittsburgh School of Medicine, Pittsburgh, USA; 2 Pediatrics, University of Pittsburgh School of Medicine, Pittsburgh, USA

**Keywords:** cyshcn, children and youth with special healthcare needs, healthcare transition, med-peds, obstructive sleep apnea, pulmonary hypertension, left recurrent laryngeal nerve palsy, ortner syndrome, charge syndrome

## Abstract

Are children’s hospitals only for children? Practically speaking, they and their associated specialty clinics often provide necessary medical and surgical care for patients older than 18 years, especially those with medical complexity. For this reason, pediatric practitioners must be familiar with both childhood-onset conditions and problems common in older and larger individuals. This case report describes a 26-year-old woman with CHARGE syndrome (coloboma/cranial nerve palsy, heart defects, atresia of the choanae, restricted development/growth, genitourinary abnormalities, ear abnormalities) who presented to a children’s hospital with hypophonia and dysphagia and was ultimately diagnosed with left recurrent laryngeal nerve palsy due to untreated sleep apnea and uncontrolled congestive heart failure leading to pulmonary hypertension that exacerbated her congenital cardiovascular abnormalities. Her hospitalization, during which she was cared for by two Internal Medicine-Pediatrics physicians (among others), exemplifies common themes in Med-Peds practice, such as a potential mismatch of expectations, experience, equipment, and policies when adults are admitted to children’s hospital, as well as an all-too-familiar lag in transitioning from pediatric to adult care for children and youth with special healthcare needs (CYSHCN).

## Introduction

Most individuals over the age of 18 years with childhood-onset complex chronic conditions in the United States receive in-patient care in adult hospitals with or without pediatric services: about 80% at 18 and at least 95% by age 21 [1]. However, medically complex adult-aged patients comprised approximately 4% of discharges at children’s hospitals before the coronavirus disease 2019 (COVID-19) pandemic, and the percentage was growing [2]. Those patients typically had congenital diseases, neuromuscular conditions, and/or technology dependence, and their in-patient stays tended to be longer and costlier than their younger counterparts' [1,2]. Therefore, clinicians at children’s hospitals should be prepared to care for individuals with medical complexity at any age or size.

I present the case of a young woman who lost her voice due to a combination of acquired cardiopulmonary pathologies and congenital anomalies attributable to CHARGE syndrome (coloboma/cranial nerve palsy, heart defects, atresia of the choanae, restricted development/growth, genitourinary abnormalities, ear abnormalities; OMIM #608892). Like many patients diagnosed before *CHD7* gene mutations were linked to most cases in 2004, she was diagnosed clinically. The birth incidence of CHARGE is one in 10-15,000 [3,4]. 

This case report was previously presented as a poster at the Mid-Atlantic Regional Meeting of the Society of General Internal Medicine on November 13, 2020. It has been expanded to include a literature review of cardiovocal syndrome and thoughts on transitions of medical care from pediatric to adult settings gathered over the author’s seven years of Med-Peds practice.

## Case presentation

A 26-year-old woman with CHARGE Syndrome, cervical myelomeningocele status post closure in infancy, Chiari II malformation, and hydrocephalus status post ventriculoperitoneal shunt, history of cleft lip and palate status post repair, and tracheostomy for subglottic stenosis status post laryngotracheal reconstruction and trach reversal presented to a tertiary children’s hospital in late December with hypophonia and dysphagia for four weeks. She was admitted to a general pediatric hospital team whose daytime and nighttime senior residents both happened to be Internal Medicine-Pediatric trainees.

Due to the whispery quality of the patient’s voice and lack of familiarity with her medical history, the young woman generally deferred to her mother as her informant. History was notable for bone-anchored hearing aid (BAHA) revision two months prior and a bout of aspiration pneumonia one month prior. Review of systems was notable for recent 7 kg weight gain, dyspnea on exertion, peripheral edema, and new nocturnal supplemental oxygen requirement. She denied coryza, odynophagia, and palpitations. She had never smoked. According to her mother, she was supposed to be on a diet of thickened liquids but rarely complied. She had been placed on furosemide 40mg once or twice a day eight months prior, in April, for dyspnea on exertion and peripheral edema; records from her adult cardiologist in a different hospital system were not readily available.

Presenting vital signs were temperature of 37.2 C, heart rate of 89 bpm, respiratory rate of 22 bpm, blood pressure of 109/58, and oxygen saturation of 97% on room air while awake and sitting upright. Physical exam was notable for a young woman with left-sided facial droop with smile (baseline); microtia and bilateral BAHAs with well-healed surgical incisions; no cervical lymphadenopathy or thyromegaly; distant heart sounds but regular rate and rhythm without a gallop; unlabored respirations, diminished breath sounds in the bilateral lung bases but otherwise no focality to auscultation; an obese abdomen; and 2+ pitting peripheral edema to the knees with venous stasis skin changes.

A broad differential diagnosis was entertained, including viral laryngitis, esophageal reflux, shunt malfunction, enlargement of her known cervical syrinx, vocal cord injury from intubation during her most recent surgery, goiter/thyroid nodule compression, and recurrent laryngeal nerve impingement due to cardiovascular pathology.

**Figure 1:**
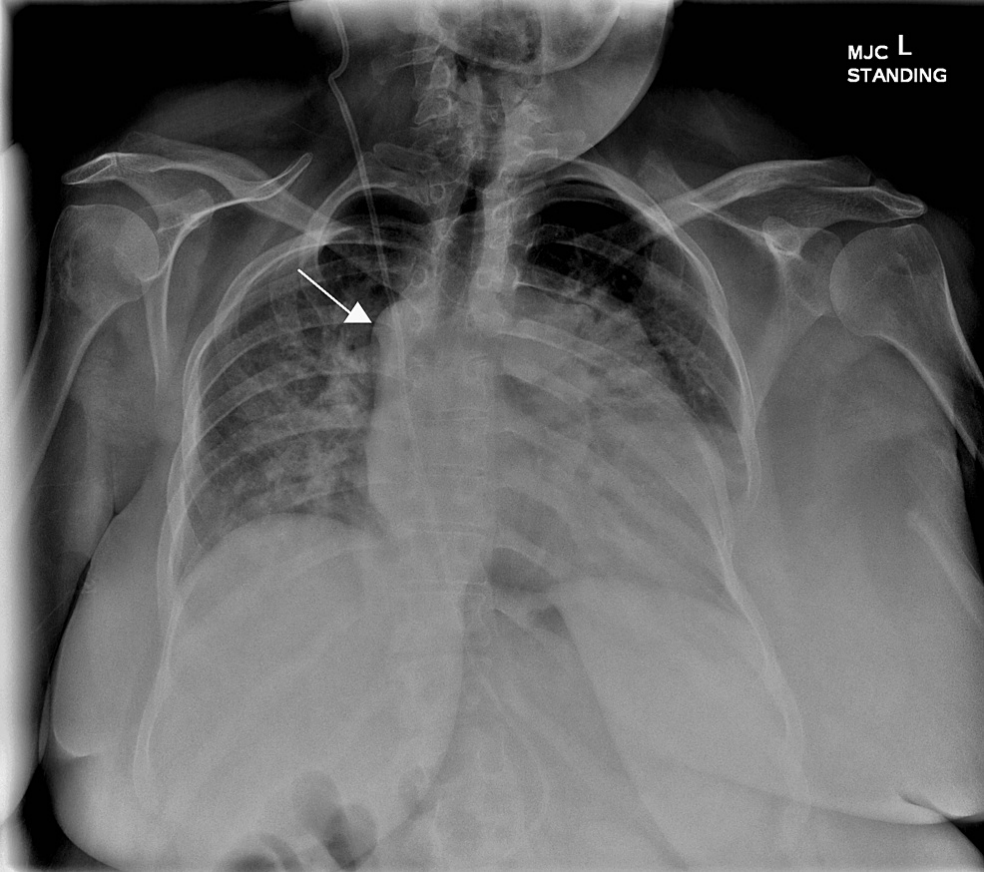
Chest x-ray on admission with pulmonary congestion In addition to the increased interstitial markings, note the intact ventriculoperitoneal shunt tubing. White arrow points to enhancement of the arch of the azygous vein where it joins the superior vena cava. Azygos continuation of the inferior vena cava has long been associated with congenital heart disease and asplenia or polysplenia (this patient has both CHD and polysplenia), but with the increased use of cross-sectional imaging, it is increasingly being recognized in asymptomatic and otherwise anatomically typical individuals.

The patient underwent an extremely thorough workup, including genetic testing for Fanconi anemia, which was negative. Laboratory testing was generally within normal limits, with the exception of a beta natriuretic peptide of 421 pg/ml. Chest x-ray hinted at her variant cardiovascular anatomy, with azygous continuation of the inferior vena cava and pulmonary congestion (Figure 1).

**Figure 2:**
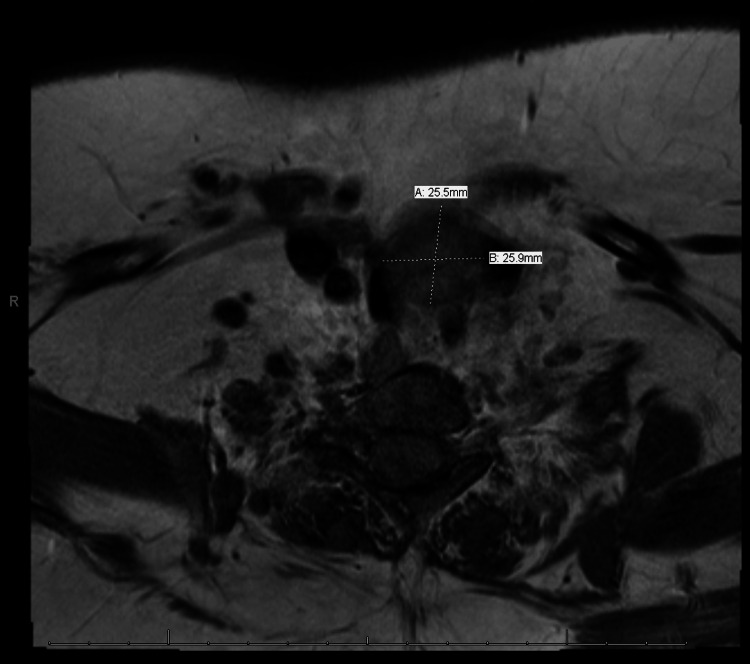
Magnetic Resonance Image of neck with 25.5mm x 25.9mm thyroid nodule.

**Figure 3:**
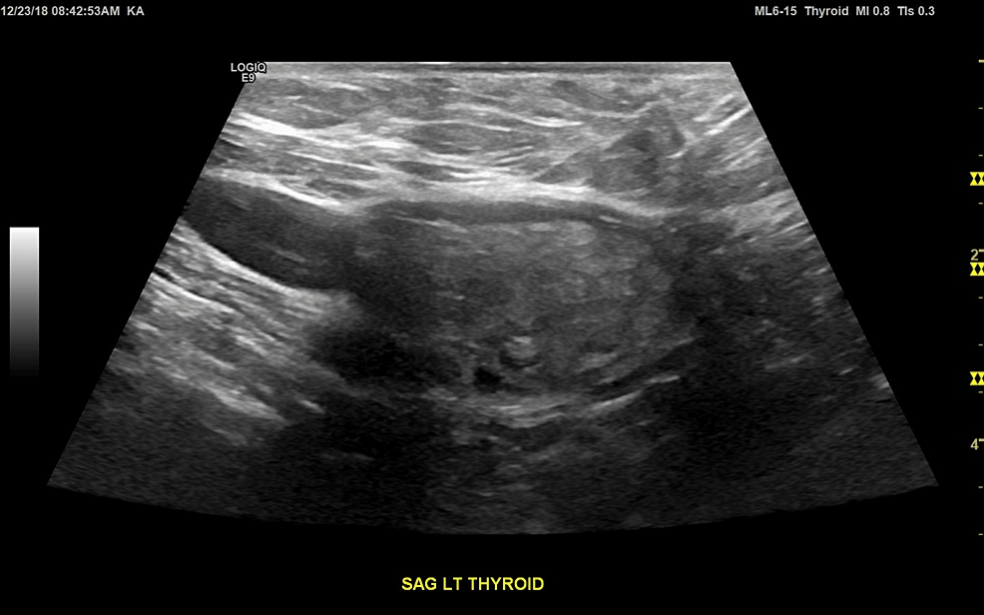
Sagittal view of the normal-appearing left lobe of the thyroid gland.

Direct laryngoscopy confirmed the left vocal cord paralysis and frank aspiration reportedly found on an out-patient scope outside of our hospital system and unavailable in the electronic medical record (EMR); there was no sign of airway trauma from her recent surgery. Shunt imaging was normal (not shown, but see Figure 1). MRI neck revealed a previously unknown 2.6 cm^2^ thyroid nodule (Figure 2). This was initially of great interest, given the path of the left recurrent laryngeal nerve, but three dedicated ultrasounds - one during this hospitalization (Figure 3) and two outpatient in the next four months - confirmed that this structure was not a discrete nodule but rather an outpouching of the left lobe of her thyroid gland, which was described as "lobulated"; the right lobe was surgically absent. Thyroid hormone studies were normal, and there were no thyroid auto-antibodies.

**Figure 4:**
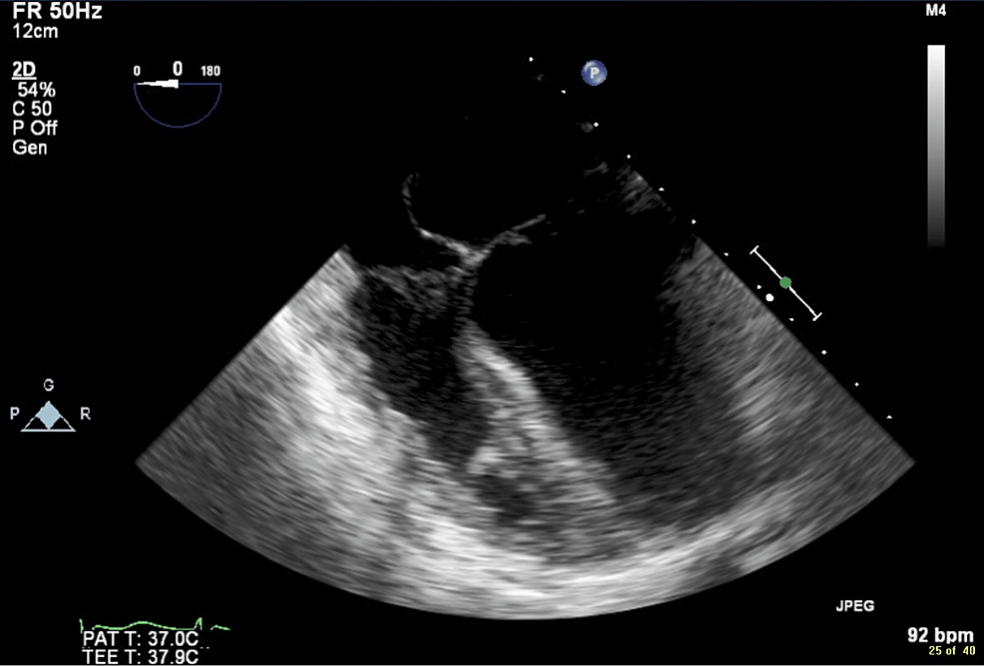
Mildly dilated left ventricle on transesophageal echocardiogram; the interventricular septum was slightly hypokinetic on motion capture.

**Figure 5:**
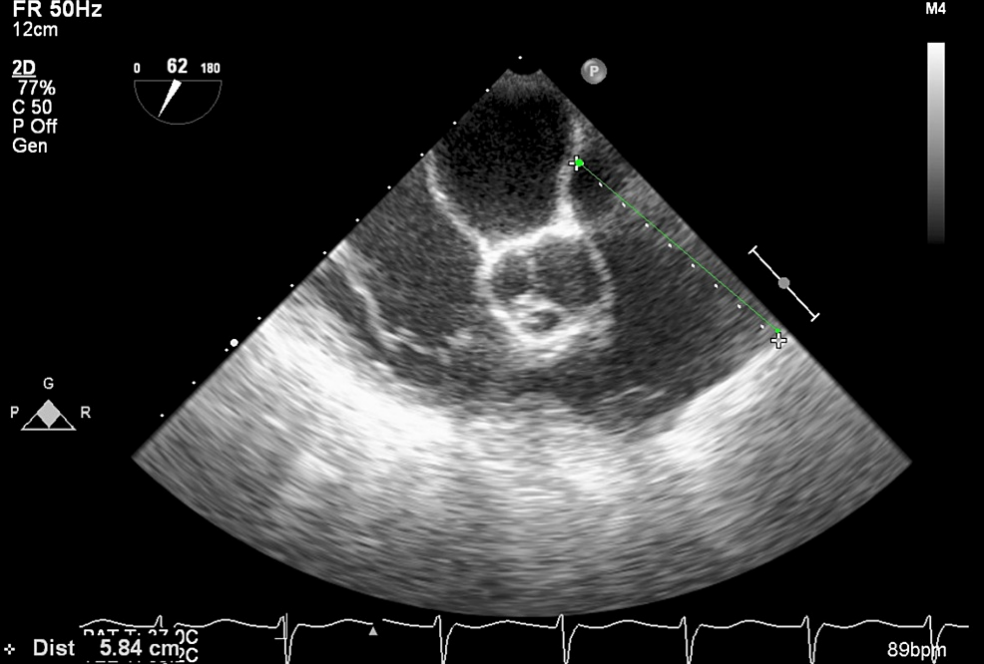
Transesophageal echocardiogram showing “severely dilated [main pulmonary artery] with estimated diameters ranging from 3.2 cm to 5.7 cm.”

Transthoracic echocardiogram (TTE) with poor image quality followed by transesophageal echocardiogram with better image quality re-demonstrated left atrial isomerism, left atrial enlargement of 4.1 cm (normal 2.7-3.8 cm), and a circumflex aortic arch with “a tight bend” (Figures 4, 5). No reliable estimate of right ventricular systolic pressure could be obtained, but the tricuspid regurgitation gradient was at least 23 mmHg (normal ≤30 mmHg). No atrial or ventricular septal defects were present.

Regarding the "tight bend" in her aortic arch, a CT chest with contrast from 10 years prior, when the patient was 16 years old, provided more detail about her unusual anatomy: “The aortic arch crosses from left to right posterior to the esophagus in keeping with a circumflex arch, the descending aorta runs just right to the midline. There is compression of the left mainstem bronchus by the descending thoracic aorta and proximal right pulmonary artery. The left mainstem bronchus measures approximately 4mm at the level of greatest compression. More proximally it measures 6.8mm and distally at the level of the hilum it measures 7.7mm. The pulmonary trunk is enlarged measuring 3.0cm, at the same level, the aorta measures 2.6cm. The pulmonary artery becomes more aneurysmal distally measuring up to 3.6cm and then tapers abruptly with the proximal left pulmonary artery measuring 1.5cm in diameter. The esophagus is also compressed at the level of the left mainstem bronchus compression. The heart itself is normal in size. No pleural or pericardial effusion is identified. There is azygos continuation of the inferior vena cava. No enlarged thoracic lymph nodes are identified.” While the report is available in the EMR, the images no longer are.

**Figure 6:**
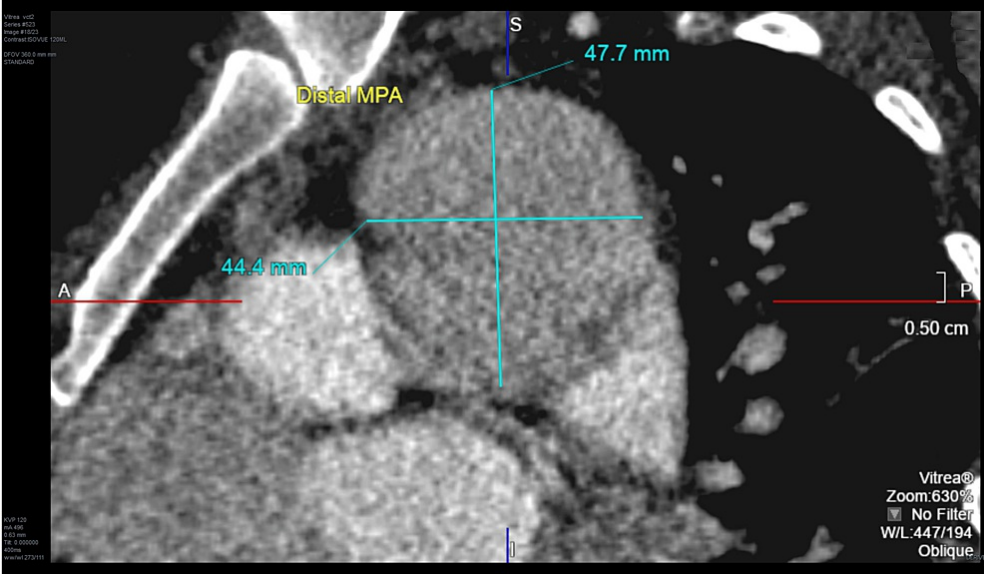
Aneurysm of the main pulmonary artery on computed tomography angiogram of the chest.

**Figure 7:**
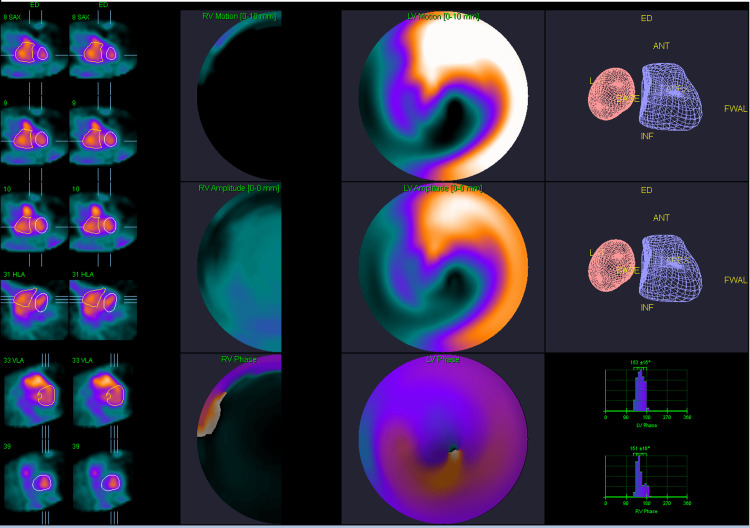
Right greater than left mildly diminished ventricular function on multi-gated acquisition scan.

CT angiogram of the chest confirmed a 4.8 x 4.4 cm aneurysm of the main pulmonary artery that had been measured as 3.6 cm^2^ nine years prior (Figure 6). Multi-gated acquisition scan (MUGA) yielded a left ventricular ejection fraction (LVEF) of 54% and a right ventricular ejection fraction of 40% (Figure 7). This was improved from an LVEF of 22% on TTE five days earlier.

Despite modest diuresis with intermittently dosed IV furosemide, she had apnea/hypopnea and nighttime desaturations that were only temporarily relieved by repositioning, such as with a neck roll. An in-home sleep study in the adult system prior to admission had found obstructive sleep apnea (OSA), and her mother divulged that she had driven an hour to bring her daughter to the children's hospital ahead of the winter holidays, because she was afraid the weather would prevent them from safely driving to a previously scheduled in-lab sleep study for initiation of continuous positive airway pressure (CPAP). Therefore, the young woman was transferred to the PICU and started on CPAP before being transferred to the heart failure service. From there she was discharged home with a new diagnosis of congestive heart failure (CHF) and instructions to follow a low-salt diet and a 2L daily fluid restriction. Medications were furosemide 40mg twice daily (BID) and losartan 50mg daily. For the OSA, she was to use a CPAP machine at 8 cm H2O + 2 L/min of supplemental oxygen at a fraction of inspired oxygen (Fio2) of 25%. For the left vocal cord paralysis, she was recommended to take nectar-thick liquids; the discharge summary did not comment on the quality of her voice.

Because her adult cardiologist preferred that the rest of her workup - myocardial perfusion spectroscopy for possible coronary artery disease and cardiac MRI to look for fibrosis - be completed in the same system in which it was started, she was re-admitted at the children's hospital twice more. Two months after the above hospitalization, she choked on pizza; TTE at that time found newly elevated right ventricular pressure of 43 mmHg + right atrial pressure. Thus the recommendation for advanced imaging was changed to left- and right-heart catheterizations. Six weeks later, in April, she was re-admitted for diuresis due to continued dietary indiscretion and lack of compliance with her diuretic regimen, which her PCP had changed at least once for documented hypotension.

Catheterization during that hospitalization confirmed the diagnosis of mild pulmonary hypertension, with mean pulmonary artery pressures of 31 mmHg on room air, 29-30 mmHg on 100% FiO2, and 29 mmHg after addition of 80 ppm inhaled nitric oxide. With pulmonary capillary wedge pressures of 15-18 mmHg, the transpulmonary pressure gradient was 11-16 mmHg. There were no anatomic causes of her pulmonary hypertension such as shunts or pulmonary vein stenosis, and there were no significant collateral vessels. At that point she was discharged on losartan 25mg BID, furosemide 40mg BID, spironolactone 50mg BID, and sildenafil 10mg three times per day; and both the primary team and the dietician emphasized the importance of a low-salt diet and fluid restriction. She was still on CPAP at 8 mmHg without supplemental oxygen. The cardiologist attending in the clinic before admission did record that both patient and mother felt that her voice quality, especially volume, was improving. A month later, with both her CHF and OSA being treated, her ENT specialist reported that her voice had returned to normal.

I would like to propose a final diagnosis of hoarseness due to left recurrent laryngeal nerve (LRLN) impingement by congenitally abnormal vasculature, exacerbated by pulmonary hypertension brought on by chronic nocturnal hypoxia from untreated OSA, in concert with undertreated CHF. Interestingly, the adult physiatrist she had met the previous August had noted, "She is on Lasix and is having a lot of edema. She is also having SOB and is on oxygen. ... She could have secondary effects from undiagnosed sleep apnea. She however has never had a sleep study because she is too scared to have one. Recommend continuing to see pulmonology." It had taken one year from symptom onset to diagnosis to resolution.

## Discussion

Presenting symptoms of recurrent laryngeal nerve palsy (RLNP) may include coughing, choking, or fatiguing while feeding due to dysphagia, as well as dysphonia ranging from alteration of usual voice quality to a weak cry (in infants) to complete loss of the ability to phonate. The recurrent laryngeal nerves innervate all the intrinsic muscles of the larynx, except the cricothyroid muscles, and are responsible for phonation and for protecting the airway during swallowing. They also carry sensory information from parts of the larynx, trachea, and esophagus. While the right recurrent laryngeal nerve loops around the right subclavian artery, the left one branches off of the vagus nerve (CN X) and wraps around the aorta, before both travel caudally between the trachea and the esophagus.

The left recurrent laryngeal nerve is vulnerable to impingement or stretching where it passes through the “aortic triangle,” where the ligamentum arteriosum connects the aortic arch to the left pulmonary artery [5]. This space is sometimes called the “aortopulmonary window,” not to be confused with the rare heart defect also called an “aortopulmonary septal defect” that involves a communication between the aorta and the pulmonary arterial trunk.

Hypophonia with or without dysphagia due to cardiovascular pathology is called cardiovocal syndrome and was previously known as Ortner syndrome. Austrian physician Norbert Ortner (1865-1935) described two cases of recurrent laryngeal nerve palsy in 1897 [6]. The first case was a 17-year-old mechanic who had had rheumatic fever at seven years old and later presented with cyanosis, anasarca, and hoarseness. Ortner initially postulated that a chronic mycotic aneurysm of the aorta could be responsible for the left recurrent laryngeal nerve paralysis, but autopsy found 2 cm of axonal damage due to thrombotic left atrial enlargement in the setting of severe mitral stenosis. The second case was the 34-year-old wife of a tailor’s assistant who had contracted rheumatic fever at 22 years old and now had two years of progressive heart failure and paralysis of the left vocal fold on laryngoscopy. After she too died, he was able to confirm his suspicion that she had stenosis of the mitral and aortic valves, that her left atrium was “almost as large as a fist” [author’s translation], and that the left recurrent laryngeal nerve showed signs of degeneration upon microscopy. Ortner suggested other potential etiologies for RLNP, including aortic aneurysm, mediastinal mass, pericardial exudate, and a case he had seen with a large pleural effusion.

Today, the etiologies of cardiovocal syndrome are recognized to be diverse, beginning with anything that closes the aortopulmonary window: left atrial enlargement (LAE) due to mitral valve stenosis or prolapse with regurgitation, LAE from left heart failure, aortic aneurysm, pseudoaneurysm after repair of coarctation of the aorta [7], and aortic dissection [8]. As Ortner suspected, the left recurrent laryngeal nerve can be trapped by aortic enlargement due to mycotic aneurysm [9]. In addition, the nerve can be damaged by mechanical insults (e.g. manipulation during catheter procedures) [10], ischemia (e.g. if the vascular supply is disrupted during intrathoracic surgery), electrical disruption (e.g. during defibrillation), and/or heat (e.g. during ablation for atrial fibrillation) [11].

Recently, enlargement of the pulmonary arterial trunk or its left branch due to all the etiologies of pulmonary hypertension has received more attention. These include primary pulmonary hypertension [12], pulmonary hypertension secondary to chronic obstructive pulmonary disease (COPD) [13], recurrent pulmonary emboli [14], and congenital anomaly of the pulmonary vasculature and lung development [15]. To my knowledge, this is the first case of cardiovocal syndrome attributable to downstream effects of obstructive sleep apnea. While secondary pulmonary hypertension develops in 27-30% of individuals with OSA but not left ventricular dysfunction or hypoxemic lung disease [16], it may be that pulmonary hypertension due to untreated sleep apnea is insufficient by itself to pinch off the LRLN and that a second factor such as congenitally anomalous vasculature is required. Another instance of multi-factorial, cardiopulmonary causation is a published case of mitral stenosis and bronchiectasis [17].

While rare to begin with, because it is typically associated with chronic conditions such as mitral valve stenosis and pulmonary hypertension, cardiovocal syndrome is more common in adults than in children. However, individuals with congenital vascular anomalies such as anomalous pulmonary arteries or Eisenmenger syndrome, as well as genetic conditions such as heritable pulmonary arterial hypertension [18], are also susceptible to developing LRLN impingement and dysphonia and/or dysphagia. Med-Peds providers should be aware that both patent ductus arteriosus (PDA) and PDA repair can be associated with LRLN palsy [19].

Thus, many structures can impinge on the left recurrent laryngeal nerve’s course; although LAE is classic for “Ortner syndrome,” pulmonary artery dilation is another possibility. In all cases, a good working knowledge of the patient’s anatomy is vital to making this diagnosis and can be obtained via history, chart review, and/or imaging. If the palsy is diagnosed and treated soon enough, and the nerve damage is not too severe, patients typically recover their voices, as this one did [20].

I propose this could be a case of an unusual presentation (RLNP) due to complications (pulmonary hypertension) of common cardiopulmonary pathologies (OSA and CHF) exacerbated by uncommon anatomy (atrial isomerism and pulmonary artery enlargement) attributable to the patient’s genetic condition (CHARGE). As RLNP predisposes individuals to aspiration, especially of liquids, the aspiration pneumonia one month prior to the December admission was a sentinel event. The patient was known to require a dysphagia diet, but because she had the intellectual and physical capacity to help herself to her beverage of choice (unthickened soda), aspiration remained a constant threat to her health. Her mother finally gave up trying to enforce thickened liquids and used the speech-language pathologist's recommendation of a "chin tuck" to reduce aspiration risk. While she continues to carry a chart diagnosis of left vocal fold paralysis, she has not had repeat laryngoscopy. She later passed a modified barium swallow but has been hospitalized for aspiration pneumonia since then.

It was difficult to ascertain from the chart the quality of our patient’s voice either before or after her hospitalization. Note writers almost never commented on whether she answered questions for herself or if her mother served as her informant, either due to cognitive deficits or lack of phonation. Med-Peds providers are probably familiar with the phenomenon of adult-aged patients with childhood-onset conditions deferring to their guardians during appointments, even for subjective questions like "How are you?", and some clinicians are guilty of assuming patients with a variety of disabilities are incapable of speaking for themselves. Happily, when the author spoke with the patient by phone in preparation for submitting this manuscript, she was able to express herself shyly but clearly.

This young woman was in the middle of transitioning to adult systems and providers, a process that can look like zigzagging back and forth between clinics and institutions. A pediatric neurosurgery physician assistant described the patient as "lost to follow up" for five years, yet she had been seen in the adult spina bifida clinic in that time frame. In the year leading up to the admission described here at length, she had seen adult cardiologists and pulmonologists but pediatric ENT surgeons. She had been admitted to both adult and children's hospitals. She had and still has a family medicine PCP, but she has since returned to the children's hospital for her cardiac, pulmonologic, and neurosurgical care. Sometimes children's hospitals are the best place for certain patients to receive care, if that is where the adults with congenital heart disease clinic is, or if equivalent care cannot be provided on the adult side. Other times change is hard for patients, caregivers, and providers alike.

Aging out of pediatric medical care is a fraught process for young adults with childhood-onset medical complexity. Patients and their caregivers are understandably hesitant to leave the healthcare professionals who have taken such good care of them or their infants and children, often literally saving their lives. They have legitimate concerns about lack of access to prior medical records; in this case, the patient's primary care practitioner and adult cardiologist and pulmonologist were part of one healthcare system, while her pediatric specialists, adult physiatrist, and the children's hospital were part of another, with only partially integrated EMRs. Even if the patient's mother had chosen for some reason to take her to one of the adult hospitals in the same system as the children's hospital, the team would have faced similar hurdles. Med-Peds providers can help if they have access to both/all EMRs. If they have been part of the community long enough, they can facilitate referrals to adult providers with whom they have personal relationships, and partner with their friends who are pediatric providers to smooth the transition.

There can be mismatch of expectations, experience, equipment, and outcomes when adults are admitted to children’s hospitals. With children’s hospitals closing (especially in rural areas) and the remaining beds filled by COVID-19 and the mental health crisis, pediatric providers typically expect young adult patients to transfer care at 19, 21, or 26 years of age. They may have less familiarity with and comfort managing “adult” conditions such as acute decompensated heart failure; in this case, despite her respiratory complaints, the patient was diuresed much more cautiously than the Med-Peds seniors were used to on the adult side. Despite the obesity epidemic affecting children and adolescents, (free-standing) children’s hospitals may not have large enough imaging devices, surgical tools, or other equipment. Indeed, our patient did not fit comfortably in the CT scanner at the children's hospital. Some young adults feel infantilized at pediatric offices and children’s hospitals, with their smaller furniture and nursery aesthetics.

Similar mismatch can occur when patients over 18 years old are admitted under internists or other adult-focused specialists with little to no exposure during training to genetic, congenital, and/or childhood-onset conditions. Empathy for patients and caregivers new to the adult medical world is no substitute for the knowledge of when and how to calculate weight-based dosing for medications for patients with restricted growth, for example. And adult hospitals are rarely built to accommodate caregivers spending the night, although young adults with developmental disabilities are hardly the only patients who benefit from having a loved one close at hand during illness. Sometimes there is confusion about why the Americans with Disabilities Act (1990) ensures the right of a nonverbal or developmentally delayed inpatient to a caregiver 24/7 when other “visitors” have to honor hospital visiting hours. Med-Peds providers may be able to explain why things work differently in children's versus adult hospitals and to "code switch" between the different medical cultures.

## Conclusions

In conclusion, a 26-year-old woman with CHARGE syndrome was admitted to a children’s hospital with four weeks of hypophonia and dysphagia. She underwent an extensive workup for potential causes of her difficulty speaking and swallowing before a final diagnosis was made of left recurrent laryngeal nerve palsy due to untreated sleep apnea leading to pulmonary hypertension that exacerbated her congenital cardiovascular abnormalities. Cardiovocal syndrome, also known as Ortner syndrome, can be caused by a variety of cardiovascular and pulmonary pathologies. Her case exemplifies common themes in Internal Medicine-Pediatrics practice - including potential mismatch of expectations, experience, equipment, and policies when adults are admitted to children’s hospitals - and a lag in transitioning from pediatric to adult care for individuals living with medical complexity from childhood. As this generation of young adults with medical complexity since childhood ages out of pediatric care and into the adult world, we will all need to know better and do better. Med-Peds physicians can lead the way.
